# The epigenetics of diabetes, obesity, overweight and cardiovascular disease

**DOI:** 10.3934/genet.2019.3.36

**Published:** 2019-08-01

**Authors:** Harem Othman Smail

**Affiliations:** Department of Biology, Faculty of science and health, Koya University Koya KOY45, Kurdistan Region-F.R. Iraq

**Keywords:** chromatin, DNA methylation, microRNAs, transcription factor, olfactory receptors, biomarkers

## Abstract

The objectives of this review were once to understand the roles of the epigenetics mechanism in different types of diabetes, obesity, overweight, and cardiovascular disease. Epigenetics represents a phenomenon of change heritable phenotypic expression of genetic records taking place except changes in DNA sequence. Epigenetic modifications can have an impact on a whole of metabolic disease with the aid of specific alteration of candidate genes based totally on the change of the target genes. In this review, I summarized the new findings in DNA methylation, histone modifications in each type of diabetes (type 1 and type 2), obesity, overweight, and cardiovascular disease. The involvement of histone alterations and DNA methylation in the development of metabolic diseases is now widely accepted recently many novel genes have been demonstrated that has roles in diabetes pathway and it can be used for detection prediabetic; however Over the modern-day years, mass spectrometry-based proteomics techniques positioned and mapped one-of a kind range of histone modifications linking obesity and metabolic diseases. The main point of these changes is rapidly growing; however, their points and roles in obesity are no longer properly understood in obesity. Furthermore, epigenetic seen in cardiovascular treatment revealed a massive quantity of modifications affecting the improvement and development of cardiovascular disease. In addition, epigenetics are moreover involved in cardiovascular risk factors such as smoking. The aberrant epigenetic mechanisms that make a contribution to cardiovascular disease.

## 1. Introduction

Eukaryotic genomes are packaged in the two types of chromatin: Gene-rich which is called euchromatin and genetically inactive which is called heterochromatin. Heterochromatin is a tightly packaged structure of DNA, and its foremost attributes is that DNA transcription is limited and Centromeres and telomeres are each heterochromatic. The euchromatin, in contrast, consists of ‘active’ chromatin: DNA sequences that are being transcribed into RNA [1]. Heterochromatin replicates in the S phase (synthesis phase) of the cell cycle later than euchromatin, most possibly keeping the DNA structure during replication. Heterochromatin additionally keeps a compact and seen structure throughout the somatic cell division (mitosis) consequently differing from euchromatin, which undergoes an ordinary cycle of condensation and unravelling at some stage in this process [2].

Epigenetics is the find out about of stable and heritable changes to the genome that can alter gene expression except altering the underlying DNA sequence. Epigenetic marks can be influenced through both the underlying genetic variation as nicely as exclusive environmental exposures [3]. Epigenetic modifications are vital for X chromosome's inactivation and genomics imprinting and indirect normal developmental programming by using imposing distinct gene expression profiles of character cell types at particular developmental levels stages [4].

Diabetes is a harmful metabolic disease characterized by way of hyperglycemia as results from defects in insulin secretion, insulin action, or both. The chronic hyperglycemia of diabetes is related to long-term damage, dysfunction, and failure of special organs, mainly the eyes, kidneys, nerves, heart, and blood vessels [5]. The two most frequenting varieties of diabetes are type 1 and type 2. Type 1 diabetes consequences of the autoimmune destruction of the pancreas's beta cells, which produce insulin. Persons with type 1 diabetes require insulin for survival in their life's; insulin may additionally be given as a daily shot or consistently with an insulin pump [6]. In type 2 diabetes (adult onset diabetes), the pancreas makes insulin, however, it either would not produce enough, or the insulin does no longer work properly. Nine out of 10 human beings with diabetes have type 2 [7]. It is usually prevalent that type 1 is the end result of gen-environment interactions, but such speedy will increase in incidence are now not explained with the aid of Mendelian inheritance. There have been several advances in our understanding of the pathogenesis of type 1. Indeed, there has been a large, wide variety of genes recognized that make a contribution to threat for this disease and several environmental factors have been recorded [8].

Obesity is a disorder characterized with the aid of extra adiposity tissue that is a supply of considerable morbidity and mortality due to quite a number weight-related with their complications. Therefore, the diagnostic contrast ought to consist of an anthropometric measure that displays elevated fat mass and an indication of the degree to which the excess adiposity is adversely affecting the health of individual patients [9]. Overweight is normally due to more body fat. However, overweight can also additionally be due to greater muscle, bone, or water. People who have weight problems typically have to a whole lot body fats [10]. The epigenome consists of DNA methylation, histone modifications, and RNA-mediated processes, and disruption of this stability may additionally cause numerous pathologies and make contributions to weight problems and type 2 diabetes (T2D) [11].

Cardiovascular disease is the main reasons of morbidity and loss of life in the Western society, accounting for a tremendous percentage of health care costs. This disease share frequent danger factor, together with obesity, lipid oxidation toxicity and low-grade inflammation, and they coexist in a super wide variety of patients [12]. The incidence of cardiovascular disease, in particular coronary artery disease and diabetes is expanded in individuals with the metabolic syndromes, a cluster of metabolic abnormalities which include central obesity, hypertension and insulin resistance [13]. Emerging proof suggests a complicated new order regulated by using epigenetic mechanisms mark cardiac cell lineage. Indeed, molecular cardiologists are in the process of shedding light on the roles performed through microRNAs, nucleic acid methylation and (histone, chromatin) changes in unique pathologies of the coronary heart [14].

## 2. Characteritics of metabolic syndrome

Metabolic syndrome is a developmental reason of morbidity and mortality worldwide. Metabolic syndrome is characterized by means of the presence of a range of metabolic disturbances along with obesity, hyperlipidemia, hypertension, and increased fasting blood sugar. Although the hazard for metabolic syndrome has mostly been attributed to an adult way of life factors such as bad nutrition, lack of exercise, and smoking, there is now robust proof suggesting that predisposition to the improvement of metabolic syndrome starts off evolved in utero [15]. Epigenetic variants have been proven to expose vulnerability to diabetes and its complications. Although it has turned out to be clear that metabolic derangements, especially hyperglycemia, can impose a long-term metabolic memory that predisposes to diabetic complications, the underlying mechanisms continue to be to be understood [16].

## 3. Epigenetic in type 1 of diabetes

Epigenetic mechanisms have an effect on gene expression that ought to predispose individuals to the diabetic phenotype for the duration of intrauterine and early postnatal development, as properly as at some stage in adult life. Furthermore, epigenetic modifications may want to account for the accelerated prices of persistent and continual micro vascular and macro vascular complications associated with diabetes [17]. An epigenetic phenomenon that is well-documented in human beings and might also be the first that springs to thinking is genomic imprinting, whereby all through germ cells development, regulatory areas of certain genes are differentially methylated and expressed relying on whether or not the gene is inherited from the mother or father [18]. Imprinting influences various genes, inclusive of some in which mutation of the expressed copy or disturbance of normal imprinting is involved in each most instances of the uncommon transient neonatal form of diabetes and, primarily based on latest evidence, curiously some cases of polygenic type 1 diabetes as properly [19]. In T1D, epigenetic phenomena, such as DNA methylation, histone modifications, and microRNAs deregulation, have been related with altered gene expression. Increasing epidemiologic and experimental evidence helps the function of genetic and epigenetic alterations in the etiology of diabetes [20].

## 4. Epigenetic mechanisms in type 2 of diabetes

Recently, epigenetic mechanisms had been proven to be concerned in endocrine cell differentiation and islet function. Genomic profiling of pancreatic islets in non-diabetic and diabetic states is wanted in order to dissect the contribution of epigenetic mechanisms to the declining proliferation potential of β cells that we see with growing old or the β-cell failure determined in diabetes [21]. Type 2 diabetes, and has been proposed to end result from altered gene regulation patterns due to epigenetic changes of developmental genes. To decide whether or not epigenetic changes may additionally play a position in the improvement of adult diabetes following Intrauterine growth regulations (IUGR), we used a rodent model of Intrauterine growth regulations (IUGR) that expresses decrease levels of Pdx1, a pancreatic and duodenal home box 1 transcription factor fundamental for β cell characteristic and development, which develops diabetes in adulthood [22].

Home box 1 transcription factor fundamental (Pdx1) is a pancreatic and duodenal home box 1 transcription factor that regulates pancreas development and β cell differentiation. Both genetic and obtained reductions in home box 1 transcription factor fundamental (Pdx1) expression in human beings and in animal models have been proven to cause type 2 diabetes, β cell dysfunction [23]. Epigenetic regulation of gene expression is one mechanism by means of which genetic susceptibility and environmental insults can lead to type 2 of diabetes. Therefore, therapeutic agents concentrated on epigenetic gene regulation can eventually be used to treat type 2 of diabetes; however, there is a lot to be learned about genome-wide epigenetic programming of health and disease earlier than these treatments can be used in theaffected person care [24].

## 5. Novel candidate gene in diabetes

There is proof that obese and diabetic human beings have a pattern of epigenetic marks unique from no obese and no diabetic individuals. The foremost long-term desires in this field are the identification and understanding of the position of epigenetic marks that may want to be used as early predictors of metabolic threat and the development of pills or diet-related treatments able to delay these epigenetic modifications and even reverse them [25]. The 14 bp INS/DEL polymorphism in the 3′UTR of HLA-G can also have an effect on the susceptibility to diabetes and coronary artery diseases (CHD), therefore suggesting a novel candidate gene. DNA hypo methylation at HLA-G promoter may additionally be a putative beneficial medical biomarker for CHD onset. Up-regulation of soluble HLA-G isoform (sHLA-G) used to be detected in prediabetic and diabetic subjects, as a result, suggesting a putative function in metabolic dysfunctions [26]. On the different hand DNA methylation of eight genes chosen based totally on a literature review of candidates doubtlessly involved in Gestational diabetes mellitus and obesogenic pathways (IGF1, IGF2, H19, ARHGRF11, MEST, NR3C1, Adiponectin, and RETN [27].

## 6. Chromatin modification in diabetes

Diabetic patients proceed to increase inflammation and vascular problems even after reaching glycemic control. This poorly understood “metabolic memory” phenomenon poses predominant challenges in treating diabetes. Recent research reveals a link between epigenetic modifications such as chromatin histone lysine methylation and gene expression. We hypothesized that H3 lysine-9 tri-methylation (H3K9me3), a key repressive and particularly steady epigenetic chromatin mark can also be involved in metabolic memory [28]. chromatin immune precipitation linked to promoter tiling arrays to profile H3 lysine-9 acetylation (H3K9Ac), H3 lysine-4 tri methylation (H3K4Me3), and H3K9Me2 in blood monocytes and lymphocytes bought from 30 DCCT traditional cure group topics (case subjects: Suggest DCCT HbA1c level >9.1% [76 mmol/mol] and development of retinopathy or nephropathy via EDIC 12 months 10 of follow-up) versus 30 DCCT intensive therapy subjects (control subjects: imply DCCT HbA1c level <7.3% [56 mmol/mol] and except development of retinopathy or nephropathy) [29].

## 7. Genes related to the overweight and obesity

Obesity and metabolic issues are growing international and are related to intelligence atrophy and dysfunction, which are danger factors for late-onset dementia and Alzheimer's disease. Epidemiological research confirmed that modifications in lifestyle, along with the customary exercise of bodily workout are in a position to stop and treat no longer only obesity/metabolic disorders, however additionally to enhance cognitive feature and dementia. Several biochemical pathways and epigenetic mechanisms have been proposed to recognize the really helpful consequences of physical workout on cognition [30]. These genes encompassed olfactory receptors (OR4D2, OR51A7, OR2T34, and OR2Y1) and quite a few downstream signaling molecules (SLC8A1, ANO2, PDE2A, CALML3, GNG7, CALML6, PRKG1, and CAMK2D), which notably regulated odor detection and sign transduction procedures inside the whole olfactory cascade, as published with the aid of pathway enrichment analyses (p = 1.94 × 10 − 10). Moreover, OR4D2 and OR2Y1 gene methylation patterns strongly correlated with each day intakes of whole energy (p < 0.0001), carbohydrates (p < 0.0001), protein (p < 0.0001), and fat (p < 0.0001) [31].

## 8. Roles microRNAs obesity and histone alteration

The involvement of histone adjustments in the development of metabolic illnesses is now broadly appreciated. Over the current years, mass spectrometry-based proteomics methods located and mapped one-of-a-kind variety of histone changes linking obesity and metabolic diseases. The listing of these modifications has been ever-growing; however, their features and roles in obesity are not nicely understood. Same as for the most properly studied histone modifications, specifically acetylation and methylation [32]. In the modern-day society, due to energy-rich diets, sedentary lifestyles, and environmental factors (such as pollution), many humans are overweight or obese [33]. According to the World Health Organization (WHO), about 39% of the world's adult population used to be overweight and around one third of them (i.e., 13% of the world's adult population) had been overweight in 2016, and the global incidence of obesity has almost tripled considering 1975. In fact, obesity is mostly preventable. Weight loss can be completed with the aid of limiting consumption of fats and carbohydrates. Many dietary interventions had been proposed to combat the obesity epidemic [34].

Recent research points out that obesity DNA and histone methylation levels, histone acetylation, and noncoding RNAs such as microRNAs (miRNAs) in oocytes and sperm. Several necessary genes, such as PPAR-α, Igf2, H19, Fyn, Stella, Sirt3, Sirt6, and Peg3 as nicely as miRNAs, such as let-7c, reportedly take part in the regulation of epigenetic changes in mammalian gametes [35]. In the remaining years, the preliminary discovery of epigenetic mechanisms represents the most applicable discovering to give an explanation for how the genome interacts with environmental factors and the ripple consequences on disease pathogeneses. Since then, all epigenetic process has been investigated via the scientific communities for almost too many years to decide which elements are concerned with this process. DNA/RNA methylation and miRNA are labeled as two of the most vital consultant lessons of such epigenetic mechanisms and deregulated activity of such mechanism can definitely make contributions to disease pathogenesis and/or development especially in tumors [36]. Male obesity might also have intergenerational and even trans generational consequences in mammals. Studies in rodents have published variations in energy metabolism and disease susceptibility in the offspring of obese males, pointing to sperm epigenetic changes as likely causal factors [37].

## 9. Link between cardiovascular disease and epigenetics

Epigenetics has been at the start studied in patients with cardiovascular disease for its outstanding role in irritation and vascular involvement [38],[39]. Furthermore, epigenetic research in cardiovascular remedy revealed a huge quantity of modifications affecting the development and progression of CVD. In addition, epigenetics is additionally worried in cardiovascular threat factors such as smoking [40].

Epigenetic mechanisms encompass DNA methylation, histone modification, and microRNA alterations, which together, allow the cell to reply rapidly to environmental changes. A quantity of cardiovascular disease Epigenetic threat factors, such as nutrition, smoking, pollution, stress, and the circadian rhythm, have been related to amendment of epigenetic cardiovascular disease Epigenetic is marks. Further examination of these mechanisms can also lead to beforehand prevention and novel remedy for cardiovascular disease Epigenetic [41]. The genetic heritability of cardiovascular disease can fluctuate significantly, relying on sex and on the situation in question. Data from some research recommend an extensive range of anywhere between about 40% to 80% genetic contribution to cardiovascular disease. Other factors regarded to have an effect on the occurrence of cardiovascular disease include the environment-gene interplay (potentially mediated by using epigenetics), variations in gene imprinting, and traits of amniotic sac development, amongst others [42]. Accumulating proof links cardiovascular getting old to epigenetic transformations encompassing a complicated interaction of DNA methylation, histone posttranslational modifications, and dynamic nucleosome occupancy ruled by means of several epigenetic factors. Advances in genomics technological know-how have led to a profound understanding of chromatin reorganization in each cardiovascular growing older and diseases [43].

Remarkably, each in utero programming and postnatal hypercholesterolemia can also have an effect on the epigenetic signature in the human cardiovascular system, thereby supplying novel early epigenetic-related pharmacological insights. Interestingly, some dietary compounds, consisting of polyphenols, cocoa, and folic acid, can modulate DNA methylation status, whereas statins might also promote epigenetic-based manage in cardiovascular disease prevention thru histone modifications [44]. Accelerated ageing, assessed by means of adult DNA methylation predicts cardiovascular disease. Adolescent accelerated getting older would possibly predict cardiovascular disease previously [45]. The rising quintessential nature of epigenetics for cardiovascular physiopathology and, importantly, the amenability to manipulation with pharmacological methods are an indication that epigenetics-based prognostic and therapeutic techniques would possibly be developed in the future [46].

## 10. Histone alteration cardiovascular disease

Chromatin is the complicated of chromosomal DNA related to proteins in the nucleus [47]. DNA in chromatin is packaged round histone proteins, in units referred to as nucleosomes. A nucleosome has 147 base pairs of DNA related to an optometric core of histone proteins, which consists of 2 H3-H4 histone dimers surrounded by using 2 H2A-H2B dimers. N-terminal histone tails protrude from nucleosomes into the nuclear lumen. H1 histone buddies with the linker DNA positioned between the nucleosomes. Nucleosome spacing determines chromatin structure, which can be widely divided into heterochromatin and euchromatin two Chromatin shape and gene accessibility to transcriptional machinery are regulated through modifications to both DNA and histone tails [48].

Histone modifications lead to adjustments in chromatin structure to render it energetic (euchromatin), in which DNA is reachable to transcriptional factors, or inactive (heterochromatin), in which DNA is inaccessible to transcriptional factors. Eight distinctive kinds of modifications catalyzed through wonderful enzymes have been described. The 2 most broadly studied histone changes are methylation and acetylation; much less properly studied modifications encompass phosphorylation, simulation, ubiquitination, ADP ribosylation, deimination, and proline isomerization [49]. In addition, miRNA performs a numerous function in the pathological manner of cardiovascular disease. Numerous researches have determined that some cardiac-specific miRNAs have achieved as certain diagnostic biomarkers and treatment goals for cardiovascular disease. The aberrant epigenetic mechanisms that make a contribution to cardiovascular disease will be discussed [50].

## 11. Conclusions

There is no doubt that histone modification and DNA methylation as two main types of epigenetics are can affect to developmental metabolic disorder and there different genes for each disease can be changes.
